# Whole plant extracts versus single compounds for the treatment of malaria: synergy and positive interactions

**DOI:** 10.1186/1475-2875-10-S1-S4

**Published:** 2011-03-15

**Authors:** Philippe Rasoanaivo, Colin W Wright, Merlin L Willcox, Ben Gilbert

**Affiliations:** 1 IMRA, Madagascar; 2 University of Bradford, UK; 3 RITAM, Oxford, UK; 4 Dept of Primary Health Care, University of Oxford, UK; 5 Fiocruz, Brazil

## Abstract

**Background:**

In traditional medicine whole plants or mixtures of plants are used rather than isolated compounds. There is evidence that crude plant extracts often have greater in vitro or/and in vivo antiplasmodial activity than isolated constituents at an equivalent dose. The aim of this paper is to review positive interactions between components of whole plant extracts, which may explain this.

**Methods:**

Narrative review.

**Results:**

There is evidence for several different types of positive interactions between different components of medicinal plants used in the treatment of malaria. Pharmacodynamic synergy has been demonstrated between the *Cinchona* alkaloids and between various plant extracts traditionally combined. Pharmacokinetic interactions occur, for example between constituents of *Artemisia annua* tea so that its artemisinin is more rapidly absorbed than the pure drug. Some plant extracts may have an immunomodulatory effect as well as a direct antiplasmodial effect. Several extracts contain multidrug resistance inhibitors, although none of these has been tested clinically in malaria. Some plant constituents are added mainly to attenuate the side-effects of others, for example ginger to prevent nausea.

**Conclusions:**

More clinical research is needed on all types of interaction between plant constituents. This could include clinical trials of combinations of pure compounds (such as artemisinin + curcumin + piperine) and of combinations of herbal remedies (such as *Artemisia annua* leaves + *Curcuma longa* root + *Piper nigum* seeds). The former may enhance the activity of existing pharmaceutical preparations, and the latter may improve the effectiveness of existing herbal remedies for use in remote areas where modern drugs are unavailable.

## Background

Over 1,277 plants belonging to 160 families were reported in 2004 to be used traditionally for the treatment of malaria following an extensive survey of the literature [1], and since then the number of species has increased substantially due to the increasing worldwide interest in anti-malarial plants. In traditional practice, several plants are often used in combination. Some of them have been screened as crude extracts for *in vitro* and/or *in vivo* anti-plasmodial activity directed to the erythrocytic stage of malaria parasites. Single active anti-plasmodial constituents have been successfully characterized from some extracts, following the pharmaceutical industry paradigm of drug discovery [2-4].

In other cases, it has not been possible to isolate active constituents from active extracts. Several explanations have been proposed for this, such as the poor quality of ethnopharmacological studies, plant material processing, preclinical laboratory protocols which are often very different from local practices, an inadequate fractionation process, degradation of active constituents during fractionation and poor biological models to demonstrate activities. Nevertheless one hypothesis that has not been extensively exploited in conventional anti-malarial therapy is synergistic interaction or multi-factorial effects between compounds present in herbal extracts [5,6]. The principal synergistic combination anti-malarials currently produced are Malarone® (atovaquone-proguanil)[7] and Quinimax® (quinine-quinidine-cinchonine)[8,9].

Pure drugs that are industrially produced or isolated from plants may be chosen for their high activity against a human disease, but they have disadvantages. They rarely have the same degree of activity as the unrefined extract at comparable concentrations or dose of the active component [10]. This phenomenon is attributed to the absence of interacting substances present in the extract. Furthermore, many plants contain substances that inhibit multi-drug resistance (MDR). A further disadvantage is that pure drugs are often more expensive to produce and distribute, and so are often unavailable and/or unaffordable to the poorest populations in remote areas who need them most. In contrast, herbal medicines can sometimes be grown and produced locally, at lower cost, by or close to those who need them [11].

The aim of this paper is to review positive interactions between components of whole plant extracts, which may explain why crude extracts are often more effective than isolated constituents at an equivalent dose. In some cases this has been exploited in the making of combined drugs. However, it is possible that a valid complementary approach is the use of standardized crude herbal medicines and/or combinations for the treatment of malaria, if their safety and efficacy are clinically demonstrated.

## The notion of synergy – evolutionary basis and mechanisms

Plants survive in a hostile environment of predatory micro- and macro-organisms in part by tough and durable external structures and by rapid growth and reproduction, but their main defences are chemical. Defensive chemicals may be present at all or certain periods of growth, and in determined locations in the plant, they may be generated after a predatory attack has been inflicted or they may be exuded into the air or soil [12]. In contrast to animals, plants do not have an adaptive immune system, so their chemical shield must cover completely the entire spectrum of macro- and micro-organisms that exist in their natural habitat and endanger their existence. However it must be recognized that groups of species growing in proximity often contribute to a collective shield [12]. The chemical defence is largely made up of relatively small molecules that fit into sites in enzymes or receptors of the predator interfering with its life processes.

Over time, the attacking organisms often develop resistance to the plant’s defences, and plants co-evolve to produce resistance inhibitors [13], resulting in a great complexity of phytochemicals in every species. Indeed if plants followed the pharmaceutical model of producing only a single compound against microorganisms which threaten them, they would probably not survive for very long. Metabolic processes in animal parasites are often mediated by enzymes and receptor molecules that possess sites which will accommodate the plant chemical defence substances, so it is not surprising that these plant defences may also serve against agents of animal diseases such as malaria.

In many cases there is evidence of synergy, but the exact mechanisms have not been elucidated. Several mechanisms may also be operating in parallel. For example *Artemisia annua* (Asteraceae) is now one of the best known anti-malarial plants [14]. The use of herbal teas prepared from the dried leaves of locally grown *A. annua* is being promoted as an alternative treatment for malaria in areas where people do not have access to, or cannot afford effective anti-malarials such as artemisinin combination therapy [11]. In a recent study, extracts were obtained from fresh *A. annua* herb either by soaking the herb in water followed by wringing out the juice by hand or by pounding the fresh herb to a pulp followed by squeezing out the juice [15]. The extracts were then analysed for artemisinin concentration and tested against malaria parasites. It was found that the anti-plasmodial IC_50_ values were 6 to 18-fold lower than was expected in terms of their artemisinin content suggesting that the activity of the extracts could not be accounted for entirely by their artemisinin content. In mice infected with *Plasmodium berghei*, the pounded juice (two doses of 500 μL, 12 h apart) equivalent to 18 mg/kg artemisinin suppressed parasitaemia by 95% compared with artemisinin 30 mg/kg as a single dose which suppressed parasitaemia by 88% [15]. In mice infected with *P. berghei* given a crude ethanolic extract of *A. annua* formulated with oil in a soft gel capsule, the ED_50_ value with respect to the artemisinin content was 35.1 mg/kg whereas the ED_50_ of pure artemisinin was 122 mg/kg[16]. These results suggest that compounds in the juice and crude ethanolic extract enhanced the action of artemisinin but whether this was due to pharmacodynamic or pharmacokinetic effects (or both) is impossible to say (although in the case of the juice it could have been due to administering it in divided doses). When the soft gel capsules were given to malaria patients in doses equivalent to 217 mg artemisinin over three days the treatment was effective in reducing fever and clearing parasites but a high rate of recrudescence was observed, although this was reduced with a longer duration of treatment [16].

In pharmacodynamic synergy, a number of substances act at different receptor targets involved in the disease to enhance the overall therapeutic effect. Synergy between different constituents of extracts has been documented not only for anti-malarial activity, but also for other pharmacological activities [17,18]. In pharmacokinetic synergy, substances with little or no activity on the causative agent assist the main active principle to reach the target by improving bioavailability, or by decreasing metabolism and excretion. Other positive interactions include complementary mechanisms of action (such as immunomodulation), reversal of resistance, and modulation of adverse effects. Each of these will be discussed in turn.

## Pharmacodynamic synergy

Strictly speaking, “synergy” or “potentiation” means that the effect of the combination is greater than the sum of the individual effects. One of the best examples of anti-malarial synergy demonstrated both *in vitro* and in clinical trials is that between atovaquone and proguanil. The activity of the combination is up to eight times greater than that of the individual compounds [7,19]. Such high levels of synergy are uncommon.

One example in natural products is the synergy between the *Cinchona* alkaloids. Almost 30 alkaloids have now been described in *Cinchona* bark [20], several of which are active against *Plasmodium falciparumin vitro*, and some of which are not [21]. The four most well-known alkaloids are quinine with its d-isomer quinidine, and cinchonine with its l-isomer cinchonidine, all of which have anti-plasmodial activity. They are found in varying proportions in different barks [22]. Interestingly, quinine is not the most potent of the alkaloids: quinidine, dihydroquinidine and cinchonine all have consistently lower 50% inhibitory concentrations (IC_50_) *in vitro*[21]. The combination of quinine with quinidine and cinchonine is 2-10 times more effective *in vitro* against quinine-resistant strains (Table 1), and the mixture of alkaloids has a more consistent effect than any of the alkaloids used singly [23]. This mixture has been used clinically although no published research has investigated whether synergy also occurs in human patients.

**Table 1:** IC_50_ (ng/ml) of cinchona alkaloids *in vitro* against two clones of *P. falciparum* (cultured in erythrocytes suspended in RPMI medium supplemented with 10% AB serum and [3H]hypoxanthine) [23]

Alkaloid	UPASx1 clone^1^	UPASx3 clone^2^
Quinine	45	280
Quinidine	22	80
Cinchonine	27	130
Quinine + Quinidine + Cinchonine (equal parts)	33	25
ΣFIC (<0.5 indicates significant synergy)	1.15	0.18

1 = quinine-sensitive

2 = quinine-resistant.

**Figure 1:**
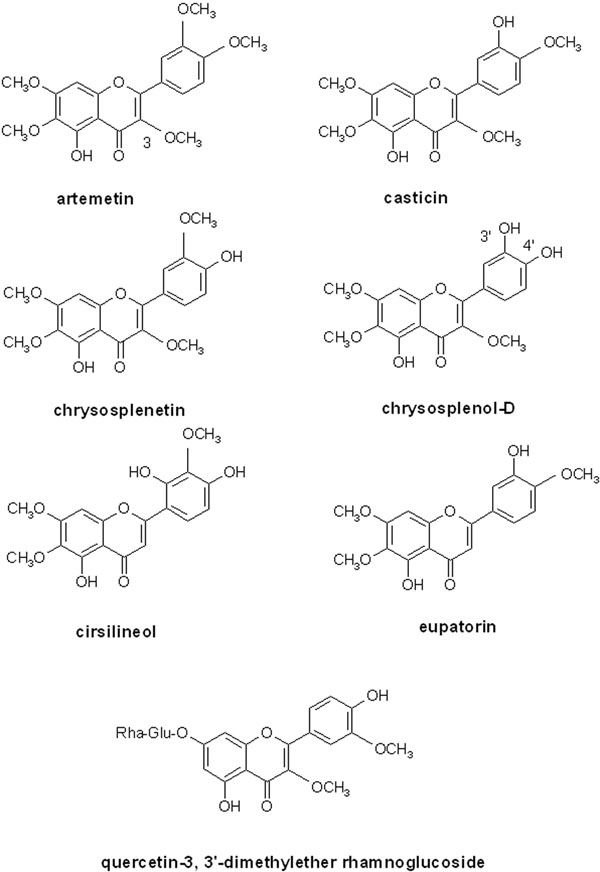
Structures of some flavonoids that contribute to the antiplasmodial properties of *Artemisia annua* and/or *Bidens pilosa*

Although it is recognized that the dose of artemisinin contained in herbal preparations is small compared to clinically recommended doses [24-26], it is widely believed that other compounds present in *A. annua*, especially flavonoids, may act synergistically to enhance the action of artemisinin [27-29]. The flavone casticin enhances the *in vitro* activity of artemisinin by 3-5 fold [28]. Some other hydroxy- polymethoxy flavone constituents (artemetin, chrysosplenetin, chrysosplenol-D, cirsilineol, eupatorin – see Figure 1) also produce enhancement of 1.1-2.2 fold but in further work, casticin was less active than in the first study (enhancement 1.3-fold) [27]. These results are perhaps unexpected given the well established antioxidant and free radical scavenging activities of flavonoids [30] and also that the mode of action of artemisinin depends on the formation of free radicals as a result of the interaction of haem iron in malaria infected red blood cells with the endoperoxide moiety of artemisinin [31]. Theoretically, it might be expected that free radicals arising from artemisinin would be effectively scavenged by *Artemisia* flavonoids thus preventing them from causing damage to the parasite. Possible reasons why the above flavonoids do not antagonize artemisinin *in vitro* may include difficulty in accessing the parasite food vacuole (in contrast to basic anti-malarials, such as chloroquine which readily accumulate into the acidic vacuole, the phenolic nature of flavonoids may impede entry due to their acidic nature). Also, the above flavonoids may be relatively weak antioxidants since they do not have some of the structural requirements associated with high antioxidant activity in flavonoids. These include the presence of a catechol group in ring B and a hydroxyl group in position 3 [30,32]. As shown in Figure 1, artemetin, casticin, chrysosplenetin and cirsilneol do not have either of the above features, although eupatorin has a 3-hydroxy group and chrysosplenol D possesses a 3’, 4’- catechol moiety.

In the Total Oxyradical Scavenging Capacity (TOSC) assay artemetin was found to have weak activity (relative TOSC value of 0.28 compared to the vitamin D analogue, Trolox) [33], while casticin was shown to have a marked inhibitory effect on lipid peroxidation (IC_50_ = 0.049 mM compared to 0.703 mM for the positive control ascorbic acid), but it was inactive in the 1,1-diphenyl-2-picrylhydrazine (DPPH) radical scavenging assay [34]. Further work to clarify the mechanisms by which these flavonoids enhance the anti-plasmodial action of artemisinin and to determine the effects of other *A. annua* constituents, especially those with high antioxidant/radical scavenging activities would be worthwhile.

In addition to at least 46 flavonoid constituents, *A. annua* also contains other phenolic compounds including coumarins and phenolic acids [35] and the contribution of all of these in combination may be important for anti-malarial efficacy. The total antioxidant capacity of *A. annua* leaf and inflorescence extracts (oxygen radical absorbance capacity, ORAC) has been reported to be remarkably high (1,125 and 1,234 μM Trolox equiv/g) and among the four highest values reported for medicinal plants [35].

**Figure 2:**
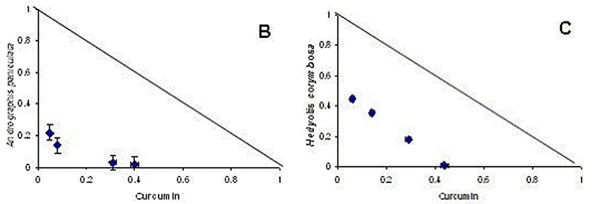
Isobolograms of the *in vitro* interaction of curcumin with methanol extracts of *Andrographis paniculata* (B) and *Hedyotis corymbosa* (C), in a chloroquine-resistant strain of *P. falciparum* (MRC-pf-303) [41]. The straight diagonal line represents the null hypothesis if there were no interaction.

There are also reports of synergy between extracts of different plants which are traditionally combined, but the mechanisms of the synergy have not yet been clarified. Curcumin (a polyphenolic compound from turmeric, i.e. *Curcuma longa* root) has direct anti-malarial activity [36,37] and turmeric is reported as a component of traditional remedies for malaria and fever in India and Samoa [38,39]. In combination with artemisinin, curcumin prevents recrudescence of malaria parasites and death in animal models[40]. In combination with *Andrographis paniculata* and *Hedyotis corymbosa* extracts, curcumin displayed a clear synergistic effect *in vitro* using the isobologram method (fig 2), and also *in vivo* in rodent malaria models [41].

Synergy has also been demonstrated *in vitro* between extracts of *Mitragyna inermis*, *Nauclea latifolia*, *Guiera senegalensis*, and *Feretia apodanthera*, traditionally used in Mali to treat malaria. Individually these have potent *in vitro* anti-malarial effects, and two combinations showed greater than two-fold synergy and absence of cytotoxicity, namely the methanol extract of *Feretia apodanthera* + ursolic acid (from *Mitragyna inermis*), and the crude alkaloids of *Mitragyna inermis* + tetrahydroharman (alkaloid constituent of *Guiera senegalensis*) [42].

**Figure 3:**
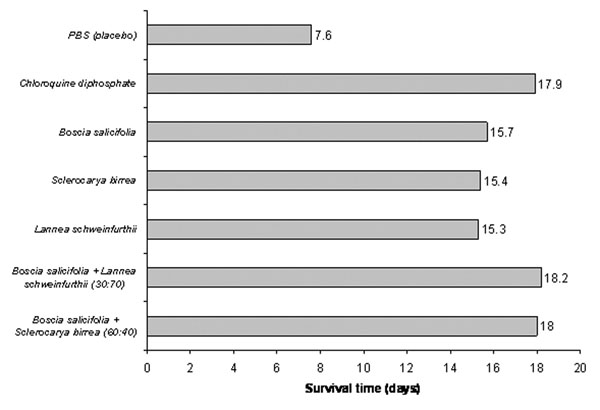
*In vivo* anti-malarial activity of single and combined plant extracts in mice infected with *Plasmodium berghei*, treated with aqueous extracts orally, at 100mg/kg [43].

Combinations of Kenyan anti-malarial plants have also shown synergy, both *in vitro* and *in vivo*[43,44]. When tested in mice, the survival time achieved by chloroquine could not be matched by any single plant extract, but was matched by two combinations of plant extracts (Figure 3). However some other combinations demonstrated antagonistic effects and reduced survival time, implying increased toxicity, which was not predicted *in vitro*. The combinations were devised by the researchers and did not represent traditional formulae. These findings demonstrate that combinations can be useful, but equally can be harmful. It is important to research combinations in traditional use, and not to assume that all combinations are synergistic.

## Positive pharmacokinetic interactions

Bioavailability and metabolism of medicinal substances can be dramatically affected by other plant constituents. There are many possible mechanisms, including an increase in permeability of the *Plasmodium* membrane to anti-malarial substances, inhibition of pump mechanisms for eliminating the drugs, modification of absorption, distribution, metabolism and excretion of active constituents. Metabolism may be inhibited in phase I (mediated by cytochromes which often oxidize foreign substances) and phase II (mediated by enzymes that derivatize the oxidized substance so that it is rendered harmless and excreted) [5,17,45].

Curcumin alone has poor oral bioavailability due to glucuronidation in the small intestine. Piperine from black pepper (*Piper nigrum* seeds) enhances the bioavailability of curcumin by 2000% in humans, due to an inhibition of this glucuronidation and slowing the gastrointestinal transit [46]. Black pepper is used as a component of herbal anti-malarial remedies in India [39,47-49]. In the same way piperine improves the bioavailability of epigallocatechin gallate (EGCG) which may improve its activity as a multi-drug resistance inhibitor *in vivo*[50] (see also below the section on MDR inhibitors).

Altered metabolism is exemplified by cytochrome P450-CYP3A4 which is involved in the metabolism of digoxin, warfarin, statins, methadone, HIV protease inhibitors, steroids, macrolides, cyclosporin, and many other drugs [51]. This cytochrome is inhibited by grapefruit juice (with its bergamottin and hydroxy dihydro bergamottin) [52], curcumin [53], garlic [54] and red wine, which may cause large increases in plasma levels of the drugs. Interestingly grapefruit juice (*Citrus paradisi*) is used to treat fever in Nicaragua [55] and garlic (*Allium sativum*) is used as a component of anti-malarial remedies in India [39,49].

Cytochrome P450-CYP2B6 is involved in the human liver metabolism of artemisinin and CYP3A4 may also contribute particularly in individuals with low CYP2B6 expression [56]. In addition, artemisinin appears to induce its own metabolism [57]. Some of the many phenolic constituents of *A. annua* may also inhibit these enzymes and so enhance the action of artemisinin *in vivo*. An important problem with the use of artemisinin as monotherapy in the treatment of malaria is the high rate of recrudescence which appears to be related to the short half-life of this drug [58]. Inhibition of metabolism of artemisinin leading to an increased half-life could theoretically result in a reduction of recrudescence. In healthy volunteers grapefruit juice doubles the oral bioavailability of artemether [59], although this combination has never been tested in malaria. Curcumin inhibits both CYP2B6 and CYP3A4[53]_,_ which may partly explain the fact that, in combination with artemisinin, it completely prevents recrudescence of malaria parasites and death in animal models [40].

The absorption of artemisinin may also be modified by other constituents of *A. annua*. In a pharmacokinetic study in healthy volunteers given a herbal tea prepared from the dried leaves of *A. annua*, artemisinin was absorbed rapidly and maximum plasma concentrations were reached 30 minutes after ingestion whereas other studies reported maximum levels more than two hours after the administration of artemisinin capsules [60]. These data suggest that artemisinin is more rapidly absorbed from the herbal tea than from pure artemisinin capsules, although the dose of artemisinin was much less than that in the capsules (95 mg and 500 mg respectively); however, no difference was seen in the bioavailability of the two preparations. The extraction of artemisinin in herbal teas has been shown to be better than would be expected taking into account its poor aqueous solubility and this appears to be due to solubilizing effects of other constituents, which may also explain the faster absorption of artemisinin seen with the herbal tea.

## Complementary mechanisms of action

Investigations on anti-malarial plants concentrate mainly on killing the parasites but rarely consider other mechanisms. Many anti-malarial herbal remedies may exert their anti-infective effects not only by directly affecting the pathogen. At least part of their effect may be indirect, by stimulating natural and adaptive defense mechanisms of the host. The immune system of the host plays a major role in complete suppression or elimination of the pathogens [61]. Extracts or single compounds that can stimulate innate and/or adaptive immunity may be able to contribute to prophylaxis and treatment not only for malaria, but also for diverse viral, bacterial, parasitic and fungal diseases [62,63]. Unfortunately, little work has been devoted to the use of these drugs for the prophylaxis and treatment of malaria. Picroliv, a standardized fraction isolated from the ethanol extract of the root and the rhizome of *Picrorhiza kurroa* (Scrophulariaceae) has been reported to activate the host immune system. Co-administration of Picroliv was found to enhance efficacy of chloroquine against experimental murine malaria [64]. A traditional Chinese mixture known as Juzen-taiho-to suppressed murine malaria by increasing the production of antibodies and interferon gamma [65].

The pathology of cerebral malaria is generally considered to be primarily of immunological origin [66]. Several compounds with known effects on the immune system were tested in a murine model of cerebral malaria [67]. Of the compounds tested, it was found that only curcumin and the synthetic Rho kinase inhibitor fasudil had significant effects on the progression of the disease. Although neither drug caused a reduction in parasitaemia, survival of the treated mice was significantly increased, and the development of cerebral malaria was either delayed or prevented. The authors concluded that an immunomodulator efficient in preventing cerebral malaria should be administered together with anti-plasmodial drugs, rather than singly, to prevent severe malaria disease [114]. However there is clearly a great difference between murine and human malaria, and immunomodulatory effects have not yet been tested clinically.

## Multidrug resistance inhibitors

Multidrug resistance is a phenomenon which usually involves a protein molecule or “pump” that straddles the cell membrane and which captures from the cytosol, often by lipophilicity, a foreign substance such as the anti-malarial agent. Powered by ATP, the pump then undergoes a conformational change which throws the entering substance out of the cell. “P-glycoproteins”are one class of such pumps.

Some herbal anti-malarials such as *A. annua* have been used for several thousand years, but resistance does not seem to have appeared at a significant level. In contrast, most isolated and synthetic anti-malarials have suffered from the appearance of resistant strains of *Plasmodium.* Resistance to isolated artemisinin in *Plasmodium yoelii* was detected at a very early stage [68] and the first signs of artemisinin resistance are appearing in *Plasmodium falciparum* from patients in Cambodia [69].

It has been shown that a number of drugs such as verapamil and antidepressants can inhibit export of chloroquine from *P. falciparum*[70-72]; they provide evidence of mechanisms that might occur with natural anti-malarials or in mixed natural-non natural combinations. Two of the *A. annua* flavones, chrysosplenol-D and chrysosplenetin, have been shown to inhibit MDR in a quite distinct microorganism, multi-drug resistant *Staphylococcus aureus*[73]. Structurally, these flavones have as substituents 3 to 4 methoxyl groups, and 1 to 2 hydroxyls but other substitution patterns may confer activity. *Bidens pilosa* also contains a flavone (quercetin-3, 3’-dimethylether rhamnoglucoside, see figure 1) that contributes to the anti-malarial activity of the plant, otherwise largely ascribed to polyacetylenes [74,75]. *Bidens pilosa* extract reverses resistance to chloroquine [76]. Epigallocatechin-3-gallate (EGCG), the most abundant tannin in green tea, is reported to be an MDR inhibitor [77] and has anti-malarial activity which is additive when combined with artemisinin [78]. Curcumin inhibits P-glycoprotein [79] and potentiates the activity of several anticancer drugs [80]; in this context it has been found to be safe in humans even at the high dose of 8g per day [81].

Alkaloids are also involved in resistance reversal. Cinchonine is a P-glycoprotein inhibitor and a very potent MDR blocker and so to a lesser extent so is quinine itself [82,83]. Cinchonine has favourable physicochemical properties for entry into the Plasmodium cell [84], and it has low toxicity in mice and rats [82]. Cinchonine has been used to good effect in combination with chemotherapy for patients with lymphoproliferative syndromes [85] and reverses resistance to quinine *in vitro*[23]. Since similar mechanisms are involved in malaria drug resistance [72,86], cinchonine should be tested as a multidrug inhibitor to use in the treatment of malaria with any proven active drug, natural or synthetic, whose activity has declined due to resistance.

Interestingly some traditional healers have started using medicinal plants in combination with chloroquine to enhance its effect. Investigation of some such plants in Madagascar has shown that they reverse chloroquine resistance *in vitro* and in mouse models [70]. These include bisbenzylisoquinoline alkaloids of *Strychnopsis thouarsii* and *Spirospermum penduliflorum*[87], alkaloids of *Hernandia voyronii*[88], and strychnobrasiline and malagashanine from *Strychnos myrtoides*[89]. Three indole alkaloids, icajine, strychnobrasiline and isoreticuline from *Strychnos icaja*, *S. myrtoides* and *S. variabilis*, which have no intrinsic anti-malarial activity, have been shown to reverse chloroquine resistance in *in vitro* tests with *P. falciparum*[90].

Very few MDR inhibitors have been tested clinically. The concept was proven in the case of chlorpheniramine reversing chloroquine resistance, but because of differing pharmacokinetics, it needed to be given for seven days [91]. More recently it has been shown that azithromycin and chloroquine act synergistically in the treatment of chloroquine-resistant falciparum malaria [92]. Azithromycin also acts synergistically with arteether in the treatment of multidrug-resistant rodent malaria [93]. A standardized extract of *Strychnos myrtoides* (containing strychnobrasiline and malagashanine) has been tested clinically in combination with chloroquine but was only given for three days, and this was not enough to reverse clinical resistance to chloroquine [94]. Because of the short half-life of these natural products, a longer duration of treatment (at least seven days) is probably necessary to produce a clinical effect.

Compounds which inhibit multidrug resistance probably exist in many more plants than are currently recognized. These compounds often have little or no direct antimicrobial effect, so would be discarded in the conventional process of screening and bioguided fractionation. However, when combined with compounds which in isolation have only moderate antimicrobial activity, they may reveal a much higher level of activity [13]. Although many plant extracts have been screened *in vitro* for anti-malarial activity, few have been tested for their ability to alter resistance mechanisms.

## Modulation of adverse effects

One of the main reasons given for isolating active compounds in the classical pharmaceutical development process is the elimination of potentially toxic but inactive compounds. Of course, it is important to assess the toxicity of whole plant extracts, but in many cases the whole extracts are not toxic. For example crude extracts of *A. annua* and *Argemone mexicana* are very safe at therapeutic doses.

The assumption that active compounds are non-toxic, and that inactive compounds are toxic, is often incorrect. Since the active compounds show toxicity to the microorganism they are probably more likely to be toxic to other organisms. For example in the case of *Cinchona* bark, quinine is as toxic as the other major alkaloids [95]. In humans, the average fatal dose of quinine for an adult is 8g, although deaths have been reported from as little as 6g in an adult and 3g in a child, which is only 3.3x the treatment dose (600mg three times daily in an adult). The fatal dose of quinidine may be as little as 250mg in cases of hypersensitivity, but is usually much greater [96]. As each alkaloid has a slightly different side-effect profile, and a combination permits a lower dose of each, paradoxically the combination may be less toxic than the equivalent effective dose of a single compound. A standardized mixture of cinchona alkaloids has been developed and marketed as “Quinimax”. It contains 71.4% quinine, with 18.6% quinidine and 5% cinchonine and is as effective as quinine in the treatment of malaria. Quinimax produces less cinchonism than quinine alone [9] and less prolongation of the QTc interval (on electrocardiography) than quinidine [8].

Another classic example is the development of febrifugine from the traditional Chinese remedy Changshan (containing *Dichroa febrifuga*). The pharmaceutical development process led to the isolation of the highly active anti-malarial compound febrifugine, which was impossible to use in clinical practice because of its intense emetic effect [97], and also its liver toxicity [98,115]. The traditional formulation [97] included several hepatoprotective plants such as *Glycyrrhiza glabra*[99], *Ziziphus jujube*[100] and *Zingiber officinale*[101]. Evidence from laboratory studies suggests that these plant extracts may have attenuated any hepatotoxicity from febrifugine, although this remains to be tested clinically. *Zingiber officinale* is also reputed as an anti-emetic, and the traditional formulation was anecdotally much better tolerated than febrifugine [97]. There is good clinical evidence for the anti-emetic effect of ginger especially in pregnant women [102], although it has not specifically been tested for its anti-emetic effect in the context of malaria. Nausea and vomiting are also common symptoms of malaria, which may explain the widespread use of ginger as one component of traditional remedies for malaria in Nicaragua [55], India [39,103], Sri Lanka [104] and Zambia [105].

## Discussion and conclusions

One definition of what counts as significant synergy is at least a two-fold increase in activity (ΣFIC <0.5) [106]. In the context of a herbal medicine, however, lower levels of synergy are still useful as they contribute to the overall effect. Although in theory it would be easy with high-throughput screening to test millions of combinations for potential anti-malarial synergy *in vitro*, this would miss any mechanisms which were dependent on metabolism (both pharmacodynamic synergy of metabolites, and pharmacokinetic synergy). For example dried *Quassia amara* leaf tea is much more active *in vivo* than *in vitro*[107].

Several anti-malarial phytomedicines are government approved and produced in standard dosages in different countries [108]. Two of these are combinations of plants, “Ayush-64” [109] and “Malarial-5” [110]. However these combinations were not designed on the basis of any evidence of synergy [109,110]. Other phytomedicines contain only a single plant which may contain several ingredients acting in synergy.

Although *A. annua* infusion is currently promoted as a single-plant remedy [14], it was not used alone in traditional Chinese medicine. Further work to elucidate the mechanisms involved in the interactions discussed above would be worthwhile and this may lead to the development of combinations of artemisinin with other natural molecules as anti-malarial agents, and of *A. annua* with other herbs.

There are several indications that curcumin may be a useful combination agent: it has some direct anti-plasmodial activity, additive to that of artemisinin *in vitro* and *in vivo* and synergistic with some other anti-malarial plants; it inhibits cytochrome P450 enzymes and so may prolong the plasma half-life of anti-malarial drugs; it inhibits multidrug resistance; and in animal models it seems to have immunomodulatory effects, preventing the development of cerebral malaria. Furthermore it is well tolerated even in very high doses. Turmeric and black pepper are both widely available and easily cultivated, more often as food spices than as medicines. Therefore, combinations including these would be good candidates to take forward into clinical trials. Ginger would also be a useful adjunct for its anti-emetic effect.

Cinchonine (or its source, *Cinchona* bark) is also a promising candidate for clinical trials, as it offers several mechanisms of action. As well as direct anti-malarial activity, cinchonine is a resistance reverser and probably has fewer side-effects than quinine. Whole *Cinchona* bark extracts were shown to be clinically safe and effective for the treatment of uncomplicated falciparum and vivax malaria in extensive clinical trials in the 1930s [111-113]. Although a combination of cinchonine with quinine has been shown to be safe and effective in clinical trials [8,9], it has never been assessed for the clinical treatment of drug-resistant malaria.

More research is needed on all of these individually and collectively [10]. There has been virtually no clinical research on potentially useful pharmacokinetic synergy or immunomodulation. Much more clinical research is needed on pharmacodynamic synergy, resistance reversal and attenuation of side-effects. This could include clinical trials of combinations of pure compounds (such as artemisinin + curcumin + piperine) and of combinations of herbal remedies (such as *Artemisia annua* leaves + *Curcuma longa* root + *Piper nigum* seeds). The former may enhance the activity of existing pharmaceutical preparations, and the latter may improve the effectiveness of existing herbal remedies for use in remote areas where modern drugs are unavailable.
